# Multi-dimensional roles of sodium-glucose cotransporter 2 inhibitors: beyond hypoglycemic and cardiorenal protection

**DOI:** 10.3389/fendo.2025.1641699

**Published:** 2025-09-29

**Authors:** Siyu Li, Yaqi Wang, Quan Gong

**Affiliations:** ^1^ Department of Immunology, School of Medicine, Yangtze University, Jingzhou, China; ^2^ National Clinical Research Center for Metabolic Diseases, Metabolic Syndrome Research Center, Department of Metabolism and Endocrinology, The Second Xiangya Hospital of Central South University, Changsha, Hunan, China; ^3^ Clinical Molecular Immunology Center, School of Medicine, Yangtze University, Jingzhou, Hubei, China

**Keywords:** nonalcoholic fatty liver disease, neuroprotection, insulin β cell, tumor, immunity

## Abstract

Sodium glucose cotransporter-2 inhibitors (SGLT2i) have been found to have a range of benefits, including improving obesity and insulin resistance, hyperuricemia, hypertension, hyperlipidemia, and other metabolic disorders. Initially used for the hypoglycemic effects, they are now found to benefit atherosclerotic cardiovascular disease and chronic kidney disease. Additionally, SGLT2i has been found to have multiple functions, such as improving liver metabolism, affecting brain function, protecting islet β cell function, anti-tumor, and affecting immune system function. This review provides an overview of the protective effects of SGLT2i on different organs and tissues, as well as the potential mechanisms underlying the functional improvement induced by SGLT2i in recent years.

## 1 Introduction

SGLT2i are commonly used as hypoglycemic drugs in clinical settings. They work by promoting the excretion of glucose in the urine, thereby reducing glucose level in the blood. Recent studies have demonstrated that SGLT2i possesses multiple functions except hypoglycemic effects, such as lowering uric acid, blood pressure, blood lipids, and weight. Furthermore, SGLT2i can delay the progression of chronic kidney disease, reduce the risk of cardiovascular events, and lower all-cause mortality (1). These effects have also been observed in non-diabetic patients (2).

Although the targets of SGLT2i are limited compared to other drugs, its effects are systemic and can impact multiple organs. While SGLT2 is predominantly found in the kidney’s proximal tubule, studies have also found expression in the intestinal mucosa and brain, with rare expression in other tissues or organs (3). This analysis seeks to consolidate the function of SGLT2i across various organs and elucidate their underlying mechanisms, establishing a groundwork for future fundamental and applied studies on SGLT2i. To craft this review, we embarked on an extensive literature trawl through PubMed, Embase, and Web of Science, culling through articles up to May of 2025. Our search was spearheaded by a blend of key terms, such as “SGLT2 inhibitors,” “sodium-glucose cotransporter-2,” and terms like “cardiorenal protection,” “NAFLD,” “neuroprotection,” “pancreatic β-cells,” “cancer,” and “immune response.” We used a mix of controlled vocabularies (like MeSH/Emtree) and free-text keywords, cleverly weaving in Boolean operators to tie them together. This strategy was like casting a wide net, ensuring that we had a comprehensive and scientifically robust grasp of the relevant studies.

## 2 Pharmacological features and clinical overview of SGLT2i

SGLT2i form a category of oral, small-molecule hypoglycemic drugs that target SGLT2 in the kidney’s tubules, which in turn cuts down on glucose reabsorption and encourages its elimination through urine. Furthermore, these compounds spark a little natriuresis and osmotic diuresis, leading to a synergistic impact that’s akin to shifting metabolic fuel and fine-tuning blood flow—a combo that has a host of health benefits, such as lowering blood sugar, trimming the waistline, reducing blood pressure, and lowering uric acid levels (4, 5).

What they all have in common pharmacologically is that they’re super selective for SGLT2 rather than its lessor relative, SGLT2i offer convenient once-daily oral administration. They are absorbed reliably and rarely cause hypoglycemia when used alone. These drugs are primarily metabolized through glucuronidation and have minimal interaction with cytochrome P450 enzymes, reducing the risk of drug interactions (6). Furthermore, their glucose-lowering effectiveness is closely tied to kidney function; as kidney function decreases, their blood sugar-lowering effect diminishes, though their heart and kidney protection benefits are partly maintained (7).

These inhibitors aren’t just about dropping sugar levels; they’re like a Swiss Army knife for health, thanks to their multi-faceted action. They quell inflammation and oxidative stress, boost fat-burning and ketone production, and kickstart the body’s self-cleaning process, autophagy (2). This versatility is what gives them their broad-spectrum protection across different organs.

To further exemplify these pharmacological attributes, the key features of representative agents are summarized in **Table 1**.

**Table 1:** Key SGLT2 inhibitors and their pharmacokinetic profiles.

Drug	SGLT2:SGLT1 selectivity	Half-life (t1/2)	Major metabolism/elimination	Approved indications	Key notes	References
Empagliflozin	~2500:1	~12 h	UGT2B7/1A3/2B17; renal/fecal	T2DM, HF, CKD	Robust CV/renal benefit (EMPA-REG OUTCOME)	(111–113)
Dapagliflozin	~1200:1	~12–13 h	UGT1A9 → 3-O-glucuronide	T2DM, HF, CKD	CV benefit (DAPA-HF); ongoing NAFLD studies	(111, 114, 115)
Canagliflozin	~160–200:1	~10–13 h	UGT1A9/UGT2B4; minor CYP	T2DM	CV/renal benefit (CANVAS); partial SGLT1 inhibition	(111, 116, 117)
Phlorizin	Non-selective (SGLT1/2)	Short	Hydrolyzed to phloretin	None	Tool compound, not clinically used	(118)

## 3 Multidimensional roles of SGLT2 inhibitorssglt2

### 3.1 Liver

Nonalcoholic fatty liver disease (NAFLD) is a common condition among people who are obese, especially those dealing with type 2 diabetes (T2DM). If left unchecked, NAFLD can progress to a more severe form known as nonalcoholic steatohepatitis (NASH), which may eventually lead to serious liver complications like fibrosis and cirrhosis. Numerous clinical studies have reported that SGLT2i can aid in improving NAFLD progression (8–10). According to an open-label randomized controlled trial, 24 weeks of dapagliflozin treatment decreased hepatic steatosis by 8%. a15% decrease in liver fibrosis score and a 25% decline in liver enzyme levels (9). The E-LIFT study discovered that empagliflozin could decrease liver fat content and enhance alanine aminotransferase (ALT) levels in patients with T2DM and NAFLD (10). These suggest that SGLT2i may be effective in improving liver health. Some experts believe that SGLT2i can delay the onset of NAFLD by promoting weight loss and decreasing glycemia (11). However, the EMPA-REG OUTCOME trial demonstrated empagliflozin’s efficacy in lowering ALT, regardless of alterations in HbA1c or body weight (12). Additionally, the analysis of the E-LIFT study found that the loss of liver fat was not related to HbA1c improvement or weight loss (10). Therefore, SGLT2i may have other mechanisms to influence the occurrence and progression of NAFLD.

#### 3.1.1 Hepatic fat metabolism altered and deposition reduced

SGLT2i can cause a partial loss of glucose from the body, affecting energy metabolism patterns in various tissues, including the liver. SGLT2i can reduce the insulin/glucagon ratio (2) and increase insulin sensitivity, altering lipid metabolism (13, 14). Hüttl M et al. conducted a study using a In a non-overweight, pre-diabetic rat study, we examined how the drug empagliflozin affects liver metabolism. The findings indicated that empagliflozin therapy decreased both neutral triglycerides and lipotoxic diglycerides within the liver while increasing the transcription of fatty acid synthase (Fas) (15). Fas is the key enzyme of fatty acid synthesis in the liver and the determinant of the maximum ability of the liver to synthesize fatty acids. According to Teruo Jojima et al., canagliflozin significantly reduces the expression of the Fas gene, which is involved in lipogenesis, and the improvement of canagliflozin on NASH may be related to the reduction of fatty acid production (16). Additionally, research indicates that Daxigliflozin enhances the production of acyl-CoA oxidase-1 (ACOX1), the key regulatory enzyme involved in lipid catabolism.This up-regulation leads to an increase in fatty acid β-oxidation, which reduces fat deposition (17). In TallyHo mice on a high-milk-fat diet, EMPA normalized key liver metabolites, including orotate (pyrimidine synthesis) and dihydrofolate (folate/methionine pathways), and normalized dysregulated acylcarnitines in females. These metabolic benefits were absent under low-fat diet conditions, suggesting that SGLT2i-induced protection is particularly effective during states of metabolic stress. EMPA also reversed the elevation of circulating amino acids induced by lipotoxic diet and improved ketone body metabolism, underscoring its systemic regulatory potential (18).

#### 3.1.2 Inflammation and oxidative stress decreased

In the onset and worsening of NAFLD, chronic inflammation and oxidative damage are key drivers. When fat tissue becomes inflamed and the body resists insulin, it sets off a chain reaction that floods the liver with excessive fatty acids and sugar, leading to endoplasmic reticulum stress, which activates the inflammasome and leads to hepatocyte death. As hepatocellular injury signals continue to activate inflammatory cells, liver fibrosis can occur. Recent studies indicate that administering empagliflozin to obese diabetic rats can lead to decreased interleukin 6 (IL-6) expression while inducing a decrease in the expression of adipokine chemokines and chemokine receptors (19). Empagliflozin combined with dulaglutide can reduce the pro-inflammatory activation of the immune system in the liver, manifesting as a decrease in regulatory T cells (Treg), pro-inflammatory macrophages, and Kupffer cells (20). Inflammation can damage cell structure, resulting in electron leaks within the mitochondria that can generate excessive superoxide. In animal models, Ipragliflozin treatment enhances liver levels of superoxide dismutase and catalase expression.These enzymes can degrade most reactive oxygen species and reduce oxidative stress levels in the liver (21). Research has shown that empagliflozin stimulates CAMKK2, a calcium/calmodulin-dependent kinase, which in turn up-regulates the expression of anti-superoxide dismutase, thereby reducing oxidative stress and lipotoxicity (22).A recent study using db/db mice and diet-induced NAFLD models revealed that dapagliflozin and canagliflozin not only reduced hepatic steatosis and fibrosis but also modulated the hepatic immune microenvironment. SGLT2i therapy diminished liver tissue levels of M1 macrophage inflammation indicators and enhanced levels of M2 macrophage anti-inflammatory markers. *In vitro*, SGLT2i promoted M1-to-M2 macrophage polarization through metabolic reprogramming, primarily by inhibiting PFKFB3, a key glycolytic enzyme. Co-culture experiments further confirmed that macrophage-mediated crosstalk suppressed hepatocyte lipogenesis (23).

#### 3.1.3 Autophagy activated and cellular senescence inhibited

Compared to patients with isolated steatosis or normal livers, patients with NASH exhibit a decrease in autophagy in liver cells. Recent studies have demonstrated that impaired mitophagy may contribute to liver injury during NAFLD and result in the formation of giant mitochondria (24). Inhibition of autophagy leads to elevated levels of TG and LDL cholesterol, causing abnormal lipid deposition in hepatocytes and further progression of NAFLD (25). Research indicates a rise in the mRNA and protein levels linked to autophagy in mice following 5 weeks of empagliflozin therapy, apoptosis resistance markers were markedly elevated in the empagliflozin group versus controls (26). Research revealed that empagliflozin boosted autophagy in liver macrophages via the AMPK-mTOR signaling pathway in diabetic mice suffering from NAFLD. This mechanism effectively curbed inflammation driven by the IL-17/IL-23 axis, ultimately alleviating hepatic injury (27).

Recent years have seen a marked increase in focus on the connection between hepatocyte senescence and NAFLD/NASH. Studies have found that liver cells in NAFLD patients exhibit signs of aging, such as shortened telomeres, increased expression of aging markers, and altered DNA methylation patterns. Additionally, aging liver cells secrete inflammatory factors and chemokines that accelerate the aging process in neighboring cells (28). One study showed that topagliflozin could reduce the expression of cyclin-dependent kinase inhibitor p21 in a mouse model, inhibiting liver cell senescence in diabetes and obesity and delaying the advancement of NASH (29) However, research on SGLT2i’s role in slowing NAFLD progression through cellular senescence inhibition remains scarce, requiring further validation.

### 3.2 Brain

The SGLT family, the most common glucose transport receptors other than glucose transporters (GLUTs), is widely distributed in the brain. All isoforms excluding SGLT5 are present in the brain (30). A study examining the protein composition of tiny blood vessels extracted from rat brain tissue showed that SGLT2 proteins were present in both nerve cell bodies and their branching extensions across various areas of the brain. What’s more, researchers using RT-PCR techniques also identified SGLT2 expression in the human cerebellum (31). D SGLT2 receptors in the CNS have been noted, but the impact of SGLT2i on the brain remains underrecognized.

#### 3.2.1 Neuroprotective effects of SGLT2i

The study demonstrates that a history of hyperglycemia or diabetes worsens cerebral ischemia in patients (32). Yamazaki et al. administered phlorizin via intraperitoneal injection in a murine focal cerebral ischemia model. They observed that this treatment improved cerebral infarction and reduced neuron damage. Furthermore, their findings proved that phlorizin directly inhibits SGLT receptors in the brain, thereby offering protection against cerebral ischemia, independent of any improvements in peripheral blood glucose levels (33). Empagliflozin has been shown to significantly impact on reducing cerebral tissue damage after cerebral ischemia/reperfusion (I/R) injury, attributing to its ability to inhibit oxidative stress, inflammation, and down-regulate apoptosis markers (34). Recent studies indicate that empagliflozin’s neuroprotective effects likely stem from its dual action of suppressing neuronal caspase-3 protein levels while boosting both hypoxia-induced factor-1α (HIF-1α) and its downstream target, vascular endothelial growth factor (VEGF). This two-pronged mechanism appears to play a key role in shielding neural tissue from damage (35).

#### 3.2.2 SGLT2i influences neurophysiology

Sodium ions are transported across the cell membrane alongside glucose through the SGLT receptor, resulting in depolarization of the cell membrane potential and increased excitability (36). SGLT2i can prevent this effect. Additionally, SGLT2i alters energy metabolism, shifting substrate utilization from carbohydrates to fatty acid oxidation and increasing ketone body production (37, 38). Ketone bodies play a role in inhibiting abnormal neuronal firing by interacting with cellular excitatory-inhibitory processes (39). The use of a ketogenic diet for epilepsy can be traced back to 500 BC (40). The clinical observation revealed that the ketogenic diet led to a 50% reduction in the number of relapses in patients with refractory epilepsy within 3 months (41). Dapagliflozin significantly reduces seizure activity in the epileptic rat model, which may be related to the reduction of neuronal glucose metabolism and neuronal cell membrane excitability (36). However, no study currently that compares the effectiveness of the ketogenic diet and SGLT2i in reducing epileptic seizures. The impact of SGLT2i on neuronal electrophysiology and its associated mechanisms remain unclear.

#### 3.2.3 SGLT2i and cognitive function in diabetes

SGLT2i has been shown to have several benefits, including impaired cognitive dysfunction, reduced oxidative stress and inflammation, and enhanced neuronal plasticity (42, 43). Clinical studies have demonstrated that SGLT2i has the potential to improve the Montreal Cognitive Assessment Scale (MoCA) score and repetitive neuropsychological status test scores (44, 45). A mechanism study showed that empagliflozin significantly increased the concentration of brain-derived neurotrophic factor and prevented cognitive impairment in obese diabetic mice (43). Additionally, SGLT2i restores mTOR to an activated physiological state, which helps prevent the onset or progression of neurodegenerative diseases (46). Furthermore, apart from its direct impact on the central nervous system, SGLT2i also exhibits inhibitory effects on acetylcholinesterase, thereby protecting cognitive function (47). Nevertheless, a limited number of clinical trials explore the application of SGLT2i for managing diabetes-related cognitive deficits. Additional studies are needed to determine whether SGLT2i can meaningfully slow cognitive deterioration in diabetic patients. A landmark study by Kim et al., drawing from nationwide population data, offers compelling insights into this question. The research examined more than 1.34 million Korean adults aged 40+ with T2DM, ultimately analyzing a matched cohort of 359,000 patients to assess how SGLT2i use impacts the likelihood of developing Alzheimer’s, vascular dementia, and Parkinson’s. The findings revealed that SGLT2i outperformed other oral diabetes medications, showing substantially lower risks for these neurodegenerative conditions: a 19% decrease for Alzheimer’s (aHR 0.81, 95% CI 0.76–0.87), 31% for vascular dementia (aHR 0.69, 95% CI 0.60–0.78), and 20% for Parkinson’s (aHR 0.80, 95% CI 0.69–0.91). When looking at the combined outcome of all-cause dementia and Parkinson’s, the reduction reached 22% (aHR 0.78, 95% CI 0.73–0.83). Crucially, these protective effects held strong even after accounting for numerous variables—from gender and overall health status to diabetes-related complications, coexisting conditions, lab results, and other medications. Subgroup analyses further revealed that the neuroprotective effects of SGLT2i were consistent across different age groups, comorbidity burdens, and metabolic conditions, suggesting that SGLT2i may exert its beneficial impact on the central nervous system through multiple synergistic mechanisms, thereby contributing to the prevention of neurodegenerative diseases (48).

#### 3.2.4 Improves brain insulin resistance

Insulin resistance serves as the key underlying mechanism in T2DM, with cerebral insulin sensitivity acting as a major player in maintaining metabolic balance throughout the body. When the brain’s ability to respond to insulin becomes compromised, it throws off the central control of metabolic processes, ultimately influencing emotional state, mental sharpness, and behavioral patterns (49). However, owing to the existence of the blood-brain barrier, progress in researching therapeutic methods to improve brain insulin resistance has been slow. Previous studies have focused on nasal insulin delivery primarily (50). Interestingly, SGLT2i is the first drug discovered to have the potential to reverse brain insulin resistance. A randomized controlled study, it was found that empagliflozin 25mg/d treatment for 8 weeks significantly improved hypothalamic insulin resistance, thereby reducing fasting blood sugar levels and liver fat content (51).

### 3.3 Protection of islet β cells function

The impairment and decline of pancreatic beta cell function are central to the development of diabetes. Consequently, safeguarding these insulin-producing cells is critical for effective diabetes management. Extensive research has demonstrated that many glucose-lowering medications exhibit protective effects capable of supporting beta cell health (52–54).

#### 3.3.1 improve insulin sensitivity

Research indicates an enhancement in insulin responsiveness with the use of SGLT2i. Phase 3 clinical trial results of canagliflozin demonstrate its ability to significantly increase the insulin secretion index and improve insulin resistance (55). Similar improvements in insulin sensitivity have been observed with empagliflozin, dapagliflozin, and other SGLT2i (56, 57). A key point to remember is that SGLT2i don’t trigger insulin release directly. Instead, they work by promoting glucose elimination through urine, which helps alleviate the harmful impact of elevated blood sugar and lessens the strain on pancreaticβcells (58). Additionally, SGLT2i can enhance the glucose utilization rate of peripheral tissues by improving hypothalamic insulin resistance (51). Jahn et al. found that 12 weeks of empagliflozin improved insulin’s vascular effects, as evidenced by improved endothelial function, reduced arterial blood pressure, and increased microvascular perfusion in skeletal and cardiac muscle. These findings support the systemic extension of SGLT2i’s effects on insulin sensitivity from a hemodynamic perspective. This suggests that SGLT2i not only alleviate β-cell stress by lowering blood glucose levels, but also enhance overall insulin efficacy through improvements in vascular function, tissue perfusion, and hormonal signaling pathways (59).

#### 3.3.2 Increase the number of islet β cells

Beyond preserving the functionality of pancreaticβcells, SGLT2i have demonstrated the ability to boostβcell mass in preclinical studies. Research by Wei R and colleagues revealed that diabetic mice treated with dapagliflozin for six weeks exhibited significant expansion of pancreatic islets andβcell volume. Subsequent investigations suggested this growth likely stems from multiple mechanisms: stimulatingβcell replication, convertingαcells intoβcells, and facilitating the differentiation of ductal cells into functionalβcells (60). Tanday N et al. conducted a study using lineage tracing technology and found that dapagliflozin intervention effectively reduces the proportion of dedifferentiated islet β cells in mice, leading to a decrease in cell loss (61). SGLT2i has also been observed to protect the remaining islet β cells by inhibiting the infiltration of inflammatory cells in the islet tissue and preventing apoptosis (62, 63).

#### 3.3.3 Promotes glucagon-like peptide-1 secretion

GLP-1, a hormone predominantly synthesized in the gut, serves a dual role in pancreatic function. Beyond triggering insulin release from beta cells, it actively supports their growth while simultaneously mitigating cellular damage caused by oxidative stress (64). Animal studies indicate that dapagliflozin enhances GLP-1 secretion by upregulating essential enzymes for its production (60). Clinical studies have also demonstrated that SGLT2i can elevate plasma GLP-1 levels (65, 66). Since SGLT2 is hardly expressed in islet α cells, whether SGLT2i affects GLP-1 content through islet α cells is still controversial. Recent evidence suggests that SGLT2i with low SGLT2/SGLT1 selectivity increases circulating GLP-1 levels, possibly through inhibition of intestinal SGLT1 production (67). Another study discovered that islet α cells could express SGLT1 receptors, and the administration of dapagliflozin can enhance its expression level. This indicates that dapagliflozin may increase the secretion of GLP-1 by significantly up-regulating the expression of SGLT1 through an SGLT1-dependent mechanism (68). By evaluating the response of insulin and C-peptide to GLP-1 after 8 weeks of dapagliflozin intervention, it was found that dapagliflozin can increase the sensitivity of islet β cells to GLP-1 in patients with T2DM (69).

### 3.4 Anti-tumor effects

In addition to its clinically proven antidiabetic effects, SGLT2i has also demonstrated therapeutic potential against various solid malignancies (70, 71). Both *in vivo* and *in vitro* studies demonstrate canagliflozin’s suppression of prostate proliferation (72), pancreatic (73), lung (74), and hepatocellular carcinomas (71). Additionally, dapagliflozin suppresses kidney cancer cell growth (75).

#### 3.4.1 Inhibition directly

Studies have found that SGLT2 is highly expressed in pancreatic cancer (43), prostate cancer (73), breast cancer (76), and highly differentiated lung adenocarcinoma (74). SGLT2i target the SGLT2 receptor in cancer cells, effectively halting glucose absorption, which in turn slashes tumor expansion and longevity. One research paper revealed that these inhibitors stifle the spread of breast cancer cells by bringing the cell cycle to a halt in the G1/G0 phase, thanks to AMPK activation, and by prompting cell death, or apoptosis (76). Similarly, Leona Yamamoto et al. obtained similar results in lung cancer cell lines, where Canagliflozin inhibited the proliferation of lung cancer cells dose-dependently (77). SGLT2i lower glucose absorption in cancer cells, alter the tumor’s cellular surroundings, and impact the energy processes within tumor cells, ultimately leading to a delay in tumor growth. While SGLT2i inhibits the transport of glucose by the SGLT2 receptor, the transport of sodium ions is also affected. Shiho Komatsu et al. found that Ipragliflozin shuts down sodium absorption through the SGLT2 receptor and induces hyperpolarization of cancer cell membranes, and at the same time, causes mitochondrial membrane potential instability sex, leading to host cell apoptosis and necrosis (78). A major retrospective study in Hong Kong involving more than 60,000 participants used propensity score matching to compare outcomes between patients on SGLT2i and those taking dipeptidyl peptidase-4 inhibitors (DPP4i). The results showed that SGLT2i use was linked to a substantially lower risk of developing hepatocellular carcinoma (HCC) in individuals with type 2 diabetes, with a hazard ratio (HR) of just 0.42. Notably, the protective effect was even stronger among high-risk groups—such as patients with cirrhosis, advanced fibrosis, or hepatitis B or C—where the HR dropped to a striking 0.12. These findings highlight the potential of SGLT2i as a promising therapeutic option for preventing liver cancer in diabetic patients, particularly those with pre-existing liver conditions (79).

#### 3.4.2 Other mechanisms

In addition to directly inhibiting the SGLT2 receptor expressed on tumor cells, studies have found that SGLT2i can also indirectly inhibit tumors through other mechanisms. Cancer cells can increase the body’s local glucose transport to the tumor to meet their energy needs while increasing the body’s insulin resistance. The use of hypoglycemic drugs can reduce insulin resistance and lower blood sugar levels, which in turn inhibits tumor proliferation and growth (80). New research has revealed that dapagliflozin effectively curbs tumor progression in obese mice with cancer. The study, led by Ali R. Nasiri and colleagues, found that this anticancer mechanism didn’t stem from heightened ketosis or direct interference with cancer cell proliferation. Rather, the drug’s efficacy came from correcting excessive insulin levels, achieved by reducing glucose absorption and metabolism within tumors (81). A recent investigation led by David Papadopoli and his team revealed that canagliflozin’s ability to prevent cell growth is linked to its impact on glutamine metabolism, a process that remains unaffected by glucose availability or the amount of SGLT2 being expressed (82). Additionally, advanced metabolomic and proteomic analyses demonstrated that canagliflozin suppresses the growth of hepatocellular carcinoma by disrupting key metabolic pathways—specifically, electron transport chain activity, fatty acid breakdown, and DNA/RNA production. The findings suggest this drug targets cancer cell proliferation through multiple interconnected biological mechanisms (83). Jingyi Luo et al. discovered that canagliflozin can reduce the metastasis, angiogenesis, and metabolism of hepatocellular carcinoma under hypoxic conditions. This decrease is realized by preventing the formation of HIF-1α protein in reprogramming contexts, potentially by modulating the AKT/mTOR signaling pathway (84, 85).

It’s important to recognize that existing research indicates only select SGLT2i show promise against particular cancer types. More extensive studies are required to fully understand how different SGLT2i combat tumors and their precise mechanisms across various cancers. Beyond efficacy, we must also examine the safety profile and practical application of integrating these drugs into cancer therapies, while thoroughly assessing their clinical benefits.

### 3.5 Effects on the immune system

SGLT2 is known to be almost absent in immune cells (3). Thus far, the influence of SGLT2i on immune system components has been insufficiently recognized. However, recent studies have increasingly discovered the involvement of immune cells in the various mechanisms through which SGLT2i protects the heart, kidney, and liver (86), although the exact mechanism is still unclear. The following outlines the potential ways in which SGLT2i may influence immune cells.

#### 3.5.1 Affect immune inflammation

Inflammatory factors are released by immune cells under inflammatory conditions. Many diseases, such as rheumatoid arthritis, atherosclerosis, and diabetes, are associated with chronic inflammation (87, 88). Chen Xu et al. found that canagliflozin has a robust anti-inflammatory effect on human immune cells, leading to reduced production and release of IL-1, IL-6, and tumor necrosis factor-α, which may be related to canagliflozin’s inhibition of intracellular glycolysis and autophagy (89). An intervention study conducted in Iran observed that Empagliflozin not only reduced the production of pro-inflammatory factors by helper T cells but also hindered their proliferative ability (90). A recent study found that canagliflozin can directly inhibit the transcription of NLR family pyrin domain-containing protein 3 (NLRP3) inflammasome-related proteins by inhibiting the NFκB signaling pathway and can also up-regulate autophagy to affect inflammation directly (91). Clinical evidence further supports the anti-inflammatory effects of SGLT2i. A study in patients with severe heart failure showed that those treated with SGLT2i exhibited significantly reduced expression of pro-inflammatory genes and decreased immune cell infiltration in epicardial adipose tissue (EAT). Metabolomic analysis revealed an enrichment of ether lipids containing oleic acid in the EAT of the treated group, suggesting a reduced tendency toward ferroptosis, which may contribute to alleviating oxidative stress. Overall, SGLT2i exert their immunomodulatory and organ-protective effects in chronic inflammatory diseases through multiple mechanisms, including inhibition of the NF-κB signaling pathway, regulation of immune cell function, suppression of pro-inflammatory cytokine release, improvement of adipose tissue metabolism, and attenuation of ferroptosis (92).

#### 3.5.2 Affect immune cell energy metabolism

The function of immune cells is closely related to their energy metabolism. For instance, naive T cells primarily rely on aerobic phosphorylation, while glycolysis becomes the main metabolic pathway for activated T cells (93). Canagliflozin inhibits intracellular glycolysis levels, promotes autophagy, and plays an anti-inflammatory role in immune cells in mice and humans (89). The switch in the energy metabolism of immune cells occurs as a result of antigenic stimulation, which leads to metabolic reprogramming. In patients with diabetes, high glucose toxicity, inflammation, and oxidative stress can activate immune cells and alter immune metabolism (94). Results from a cohort study found abnormally enhanced peripheral blood T cell glucose uptake in patients with type 1 diabetes, which was correlated with C-peptide and HbA1c levels (95). SGLT2i can potentially reduce the abnormal activation of immune cells by improving glucotoxicity and lipotoxicity, thereby altering the energy metabolism pattern of immune cells (96). In immune thrombocytopenia, empagliflozin can modify the energy metabolism of CD4+ T cells in peripheral blood, shifting it from oxidative phosphorylation to glycolysis. The mechanism behind this effect may be associated with the inhibition of the mTOR signaling pathway by empagliflozin (97).

SGLT2i causes partial glucose loss, enhanced fatty acid oxidation, and increased ketone body production (37). In addition to being metabolites, ketone bodies can serve as energy substrates for specific cells. This utilization of ketone bodies for energy supply is a mechanism that protects cells (98). Karagiannis F et al. discovered that severe patients infected with SARS-CoV-2 experienced a decrease in the synthesis of β-hydroxybutyrate (BHB), an increase in the level of glycolysis in T cells, and impairment in the immune function of CD4+ T cells. Mechanism research revealed that BHB, acting as a surrogate dye, enhances the production of energy-donating amino acids (glutamate and aspartate) and glutathione while promoting oxidative phosphorylation to restore cellular function (99). Furthermore, it has been discovered that the ketogenic diet impacts immune function. Ketone bodies can enhance T cell reactivity and cell lysis ability by activating mitochondrial oxidative metabolism-based metabolic reprogramming, additionally regulating cell differentiation and increasing the generation of memory T cells (100). While there is currently no evidence to suggest that SGLT2i can serve as a substitute for the ketogenic diet, it is worth noting that it exhibits exceptional safety and feasibility as a ketogenic diet mimic (101). A prospective pilot study systematically evaluated the changes in mitochondrial function of immune cells before and after 6 months of SGLT2i treatment in kidney transplant recipients with T2DM, aiming to explore its potential immunometabolic regulatory mechanisms. The study revealed that SGLT2i significantly preserved mitochondrial membrane potential in lymphocytes, reduced reactive oxygen species (ROS) levels, and enhanced mitochondrial biogenesis. These improvements were closely associated with reductions in body weight and LDL-C levels. Under PHA-induced immune activation, SGLT2i notably enhanced the metabolic adaptability of lymphocytes. This study, for the first time in human transplant recipients, demonstrated that SGLT2i may participate in immunometabolic reprogramming by improving mitochondrial homeostasis and oxidative stress in immune cells, thereby offering a potential cellular and metabolic explanation for its cardio-renal protective effects. These findings expand our understanding of the “beyond glycemic control” benefits of SGLT2i and provide new insights into its application as an immunometabolic intervention in chronic diseases (102).

## 4 Key molecular pathways modulated by SGLT2i

### 4.1 Anti-inflammatory pathways

SGLT2i reliably exhibit strong anti-inflammatory properties in various disease contexts. One of their primary actions involves blocking the NF-κB signaling cascade. By interrupting this pathway, SGLT2i effectively curb the production of inflammatory markers like TNF-α, IL-6, and IL-1β (103). Additionally, these drugs directly interfere with the NLRP3 inflammasome—a key molecular assembly responsible for processing IL-1β—through two distinct mechanisms: decreasing mitochondrial oxidative stress and enhancing the autophagy-driven removal of inflammasome elements (86, 104). The resulting decline in inflammatory cytokines helps alleviate persistent systemic inflammation, which plays a central role in the development of cardiometabolic and renal disorders.

### 4.2 Metabolic adaptation and bioenergetic regulation

SGLT2i fundamentally alter cellular energy dynamics by mimicking a fasting state. These drugs trigger AMPK, the body’s master metabolic regulator, which in turn suppresses the mTOR pathway—a key promoter of cell growth and synthetic processes (105). This metabolic switch through the AMPK/mTOR pathway not only stimulates cellular cleanup but also dials down inflammation while boosting insulin responsiveness (106).

What’s more, SGLT2i rev up peroxisome proliferator-activated receptor alpha (PPARα), shifting the body’s energy preference toward burning fats and producing ketones (107). This metabolic pivot offers the heart and brain a cleaner, more efficient fuel source—especially when they’re under duress—effectively upgrading the body’s energy economy under stress (2).

### 4.3 Mitigating oxidative stress

Oxidative stress plays a pivotal role in cellular damage associated with diabetes and aging. SGLT2i significantly bolster the body’s antioxidant defenses by stimulating the nuclear factor erythroid 2-related factor 2 (Nrf2) pathway (108). Once activated, Nrf2 moves into the nucleus and binds to antioxidant response elements (ARE), triggering the production of key antioxidant enzymes like superoxide dismutase (SOD) and catalase (CAT) (109). By ramping up these protective mechanisms, SGLT2i help counteract harmful ROS, minimizing oxidative damage to vital cellular components—lipids, proteins, and DNA—and ultimately safeguarding cellular integrity (110).

### 4.4 Insulin signaling and survival pathways

Enhancements in insulin sensitivity throughout the body are largely thanks to the improved signaling process involving IRS-1, PI3K, and Akt (59). This trio plays a pivotal role in glucose absorption, protein formation, and the sustenance of cellular life. By easing glucotoxicity and inflammation, medications like SGLT2i can prevent IRS-1 from getting phosphorylated at the serine site—a move that’s a hallmark of insulin resistance (105). This in turn ensures the restoration of effective insulin signaling. Furthermore, the activated Akt route helps quash apoptosis and maintains the delicate balance of metabolism.

### 4.5 Anti-tumor mechanisms

SGLT2i don’t just impact metabolism; they also pack a punch against cancer through various mechanisms. By cutting off glucose supplies, they stop the stabilization of HIF-1α, a key player in how tumors cope with low oxygen levels. Lower HIF-1α levels then bring down the production of its targets, such as VEGF, effectively shutting down the growth of new blood vessels in tumors and preventing the spread of cancer (71). Moreover, these inhibitors hit the brakes on the Akt/mTOR pathway, which is crucial for cancer cells to multiply, survive, and make proteins (76, 84). Certain SGLT2 inhibitors even go the extra mile by directly blocking important enzymes like PFKFB3, throwing a wrench into the aerobic glycolysis, or Warburg effect, that many cancer cells rely on (23).

**Table 2** outlines the principal molecular pathways influenced by SGLT2i.

**Table 2:** Key molecular pathways modulated by SGLT2i.

Pathway	Key components	Biological effect	References
Inflammation	NF-κB, NLRP3, IL-6, TNF-α	Reduces cytokine production, attenuates chronic inflammation	(86, 103, 104)
Energy Sensing	AMPK, mTOR	Promotes catabolism, inhibits anabolism, induces autophagy	(105, 106)
Oxidative Stress	Nrf2, SOD, CAT	Enhances antioxidant defense, reduces ROS damage	(108, 109)
Insulin Signaling	IRS-1, PI3K, Akt	Improves insulin sensitivity, promotes cell survival	(59, 105)
Tumorigenesis	HIF-1α, VEGF, Akt/mTOR, PFKFB3	Inhibits angiogenesis, metastasis, and tumor metabolism	(23, 71, 76, 84)

## 5 Future perspectives and clinical implications

Even with all the hype around SGLT2i and their potential across various health areas, we’re not out of the woods yet. For starters, a lot of the buzz is based on early lab work or smaller trials. We desperately need some big, robust randomized controlled trials to really prove these drugs work and are safe for all sorts of patients. Also, we’re still fuzzy on exactly how SGLT2i tinker with things like immune function, brain protection, and even how tumors behave. More digging is definitely needed there. And let’s not forget the big question mark hanging over what these drugs do in the long run to organs other than the kidneys and heart, especially in people who don’t have diabetes.

On the bright side, the fact that we’re using SGLT2i for more than just blood sugar control shows they might be true multi-organ protectors. Looking ahead, research should focus on combining them with other treatments, like GLP-1 receptor agonists or drugs that tweak the immune system. Figuring out which biomarkers can help us pick the right patients and seeing if these drugs are actually cost-effective in the real world are also key. Putting all this together will be vital to really unlock the full potential of SGLT2i and give patients the best possible results.

## 6 Summary

**Figure 1:**
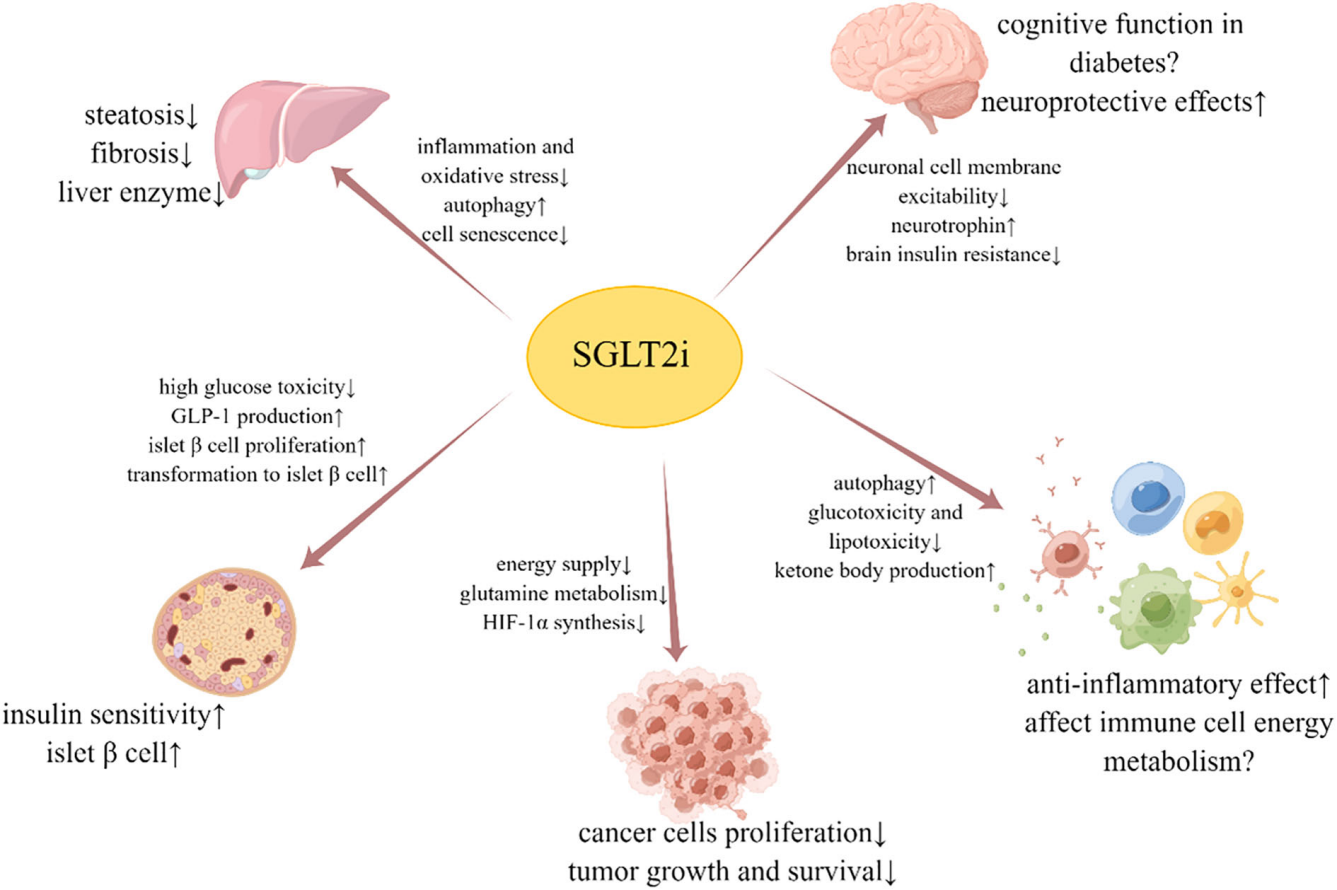
Beneficial effects and possible mechanisms of SGLT2i on liver, brain, islet β cell, tumor and immune cell.

In addition to its hypoglycemic and cardiorenal protective effects, SGLT2i also exhibits multiple mechanisms for organ protection. A simplified graphic summary of the beneficial effects and possible mechanisms of SGLT2i is provided in **Figure 1**. It can delay the occurrence and development of NAFLD by altering liver fat metabolism, reducing inflammation and oxidative stress, activating autophagy, and inhibiting liver cell aging. SGLT2i has also shown the ability to protect neuron function, influence neurophysiological activity, and improve brain insulin resistance, which is associated with cognitive function. Furthermore, studies have found that SGLT2i can safeguard islet function, slow down tumor growth, and impact immune inflammation and energy metabolism of immune cells. In conclusion, despite the limited distribution of SGLT2 receptors, the roles of SGLT2i are extensive and varied, and its multi-organ protective mechanism holds significant potential for clinical application.
